# *PtrVCS2* Regulates Drought Resistance by Changing Vessel Morphology and Stomatal Closure in *Populus trichocarpa*

**DOI:** 10.3390/ijms24054458

**Published:** 2023-02-24

**Authors:** Meng Li, Hao Dong, Jiyuan Li, Xiufang Dai, Jiaojiao Lin, Shuang Li, Chenguang Zhou, Vincent L. Chiang, Wei Li

**Affiliations:** 1 State Key Laboratory of Tree Genetics and Breeding, Northeast Forestry University, Harbin 150040, China; 2 Forest Biotechnology Group, Department of Forestry and Environmental Resources, North Carolina State University, Raleigh, NC 27695, USA

**Keywords:** *Populus trichocarpa* (Black Cottonwood), stem developing xylem, drought stress, stomata aperture, transcriptomic sequencing, chronic stress

## Abstract

Drought has severe effects on plant growth, forest productivity, and survival throughout the world. Understanding the molecular regulation of drought resistance in forest trees can enable effective strategic engineering of novel drought-resistant genotypes of tree species. In this study, we identified a gene, *PtrVCS2*, encoding a zinc finger (ZF) protein of the ZF-homeodomain transcription factor in *Populus trichocarpa* (Black Cottonwood) Torr. & A. Gray. ex Hook. Overexpression of *PtrVCS2* (*OE-PtrVCS2*) in *P. trichocarpa* resulted in reduced growth, a higher proportion of smaller stem vessels, and strong drought-resistance phenotypes. Stomatal movement experiments revealed that the *OE-PtrVCS2* transgenics showed lower stomata apertures than wild-type plants under drought conditions. RNA-seq analysis of the *OE-PtrVCS2* transgenics showed that *PtrVCS2* regulates the expression of multiple genes involved in regulation of stomatal opening and closing, particularly the *PtrSULTR3;1-1* gene, and several genes related to cell wall biosynthesis, such as *PtrFLA11-12* and *PtrPR3-3.* Moreover, we found that the water use efficiency of the *OE-PtrVCS2* transgenic plants was consistently higher than that of wild type plants when subjected to chronic drought stress. Taken together, our results suggest that *PtrVCS2* plays a positive role in improving drought adaptability and resistance in *P. trichocarpa*.

## 1. Introduction

In addition to being a source of food, fuel, and materials, forests can be used to sequester carbon dioxide through photosynthesis, affecting soils and weather around the world [1]. The vascular cambium of trees differentiates into xylem and phloem, in which xylem mainly transports water and mineral nutrients [2]. However, drought, causing loss in xylem hydraulic conductivity, critically impairs metabolic and hydraulic function [3]. When the water potential of root cells is lower than that of soil, water enters the cells in the root cortex through symplastic movement and moves into the vascular system [4]. However, under drought stress, insufficient water enters the vascular system, and gas enters a single vessel cell, forming an embolism, which travels from one vessel to another. Embolisms can cause serious damage to water transport [5]. It is usually measured by the percentage loss of conductance (PLC) [6]. The broken water column causes the water to not be transported to shoot, which can result in desiccation and mortality [7]. To cope with drought stress, trees have developed many strategies, such as reducing water loss and protecting the water transport capacity of the plant [8].

In response to the biotic stress of bacteria, fungi, viruses, and insects, plants often protect themselves by regulating some ion signals and hormone signals [9,10]. Similarly, signaling of hormones, such as abscisic acid (ABA), jasmonic acid (JA), brassinosteroid (BR), etc., also plays various roles in plant response to abiotic stress [11]. The main way water is lost in plants is by gas exchange in leaves; closing stomata is one of the most effective way to reduce water loss [12]. The most common light-induced stomatal closure is mediated by ABA [13]. ABA induces the increase of cytoplasmic calcium (Ca^2+^) in guard cells. Activation of anion channels leads to membrane depolarization and potassium (K^+^) outflow, which both promote water outflow, resulting in filling loss and thus stomatal closure [14]. Water use efficiency (WUE) is the ratio of carbon taken by plants with respect to water use. The effects of photosynthesis and stomata on WUE determine the accumulation of plant biomass [15]. Various metabolites from photosynthesis increase WUE by controlling sucrose metabolism in guard cells [16].

In addition to reducing water loss, it is important to ensure water transport in plants. The key factor is prevention of embolism. For tree species, wider vessels may lead to more embolism [17]. Polar auxin transport in the xylem to form small diameter highly aggregated vessels can alter the hydraulic properties of the stem [18]. Small-diameter vessels do not develop severe embolism or horizontal deficiency [19]. Additionally, through a complex regulatory network, cellulose, hemicellulose, and lignin are biosynthesized and deposited to thicken secondary cell walls and form wood [20]. For the long distance transport of water, lignin is deposited in the cell wall, a process that can be triggered by abiotic stresses [21]. Xyloglucan is the main hemicellulose of the cell wall, and xyloglucan endotransglycosylase/hydrolase activity is involved in xylem cell wall thickening and regulation of xylem water transport [22].

Stress signals are transmitted through hormone signals to functional genes and transcription factors involved in cell protection, including v-*myb* avian myeloblastosis viral oncogene homolog (MYB), basic leucine zipper (bZIP), zinc finger homeodomain (ZF-HD), etc., which have been proven to be involved in responding to abiotic stresses such as salt tolerance, cold tolerance, and drought tolerance [23,24]. Plant-specific ZF-HD transcription factors are induced by polyethylene glycol, sodium chloride, and cold and hot stress in wheat [25]. In *Chenopodium quinoa* (Willd.), *CqZF-HD14* regulates *CqNAC79* and *CqHIPP34* promotes photosynthetic pigment accumulation, maintains antioxidant capacity, and enhances drought resistance [26]. *DcHB30*-encoding ZF-HD protein and *DcWRKY75* are antagonistic to the regulation of petal senescence in carnation (*Dianthus caryophyllus* L.) [27]. The *PpOFP1* gene of peach (*Prunus persica* Batsch) has been allogeneously transformed in yeast and tomato to exhibit salt tolerance in interaction with *PpZFHD1* [28]. The members of the ZF-HD family in *Arabidopsis thaliana* (Heynh.) are divided into seven groups except for MINI ZINC FINGER proteins (MIFs), which are only found in seed plants [29]. *AtMIF1* is involved in the regulation of various hormones in *Arabidopsis thaliana* development, including gibberellins, ABA, auxin, ethylene, etc. [30]. In tomato (*Solanum lycopersicum* L.), SIIMA recruits SIKNU to form a complex with TOPLESS and HDA19 that binds and inhibits *SIWUS* transcription to regulate carpels and fruit size [31]. Although some ZF-HD family genes respond to drought stress [32], the function of MIFs in response to abiotic stresses has not been previously reported. Moreover, little is known about the ZF-HD subfamily in woody plants except that it contains 21 members in *Populus trichocarpa* [33].

Recently, we have identified a ZF-HD TF family gene, *PtrVCS2*, in *P. trichocarpa*. *PtrVCS2* is involved in regulating cambium proliferation by directly binding to *PtrWOX4a* gene promoter through interaction with *PtrWOX13* in *P. trichocarpa* [34]. The activity of vascular cambium also affects xylem development [35]. In this study, we revealed the role of *PtrVCS2* in regulating xylem development and stomatal closure of *P. trichocarpa* and found that overexpression of *PtrVCS2* decreases the percentage of embolism in xylem and improves drought resistance of *P. trichocarpa*.

## 2. Results

### 2.1. Overexpressing PtrVCS2 Gene Results in More and Smaller Vessels in Xylem Tissue

**Figure 1:**
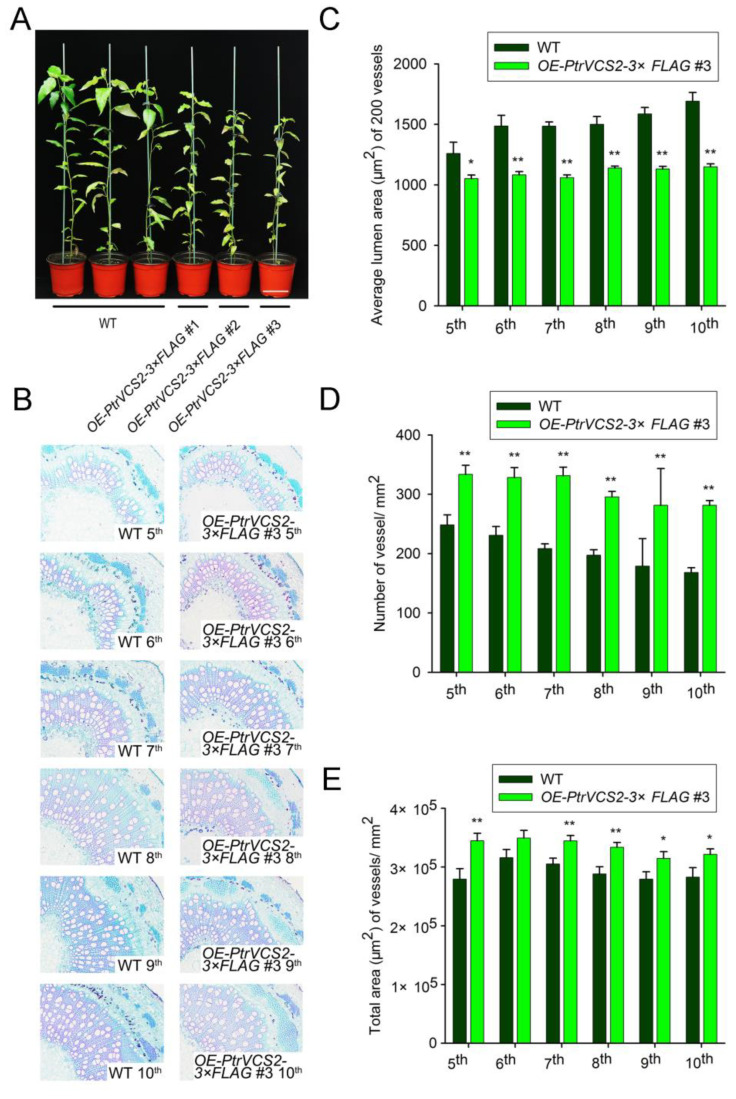
Overexpressing *PtrVCS2* gene affects the size and number of vessels in xylem tissue of *P. trichocarpa*. (**A**) The growth phenotypes of 4-month-old *OE*-*PtrVCS2-3×FLAG* #1, #2, #3 and wild-type (WT) plants. Bars = 10 cm. (**B**) Stem cross-sections of wild-type (WT) and *OE-PtrVCS2-3×FLAG* #3 transgenic plants. Bars = 200 µm. (**C**–**E**) Average lumen area (μm^2^) of 200 vessels cells (**C**), total number of vessel/ mm^2^ (**D**), and total area (μm^2^) of vessels/ mm^2^ (**E**) using vessel cells from (**B**). Error bars represent standard errors for three independent replicates with at least 200 *OE-PtrVCS2* transgenic vessel cells and 200 WT plant vessel cells for each genotype in each replicate, and asterisks indicate significant differences between the *OE-PtrVCS2* transgenics and WT plants. * *p* < 0.05, ** *p* < 0.01 (Student’s *t*-test).

We previously generated transgenic *P. trichocarpa* plants overexpressing *PtrVCS2-3×FLAG* (*OE*-*PtrVCS2-3×FLAG*) under the control of a CaMV 35S promoter (Figure 1A). The transgenic line #3 (*OE*-*PtrVCS2-3×FLAG* #3) with the highest levels of transgene overexpression was selected and propagated with the wild type (WT) and maintained in a greenhouse. Stem cross-sections analysis revealed that the xylem vessels in *OE*-*PtrVCS2-3×FLAG* #3 plants were smaller than those in wild-type plants from the 5th stem internode (Figure 1B, C) where stem secondary growth begins. The vessel number per unit area in *OE*-*PtrVCS2-3×FLAG* #3 plants was greater than that in wild-type plants (Figure 1D), which resulted in an increase in the total vessel lumen area (void area) in *OE*-*PtrVCS2-3×FLAG* #3 plants (Figure 1E).

### 2.2. Overexpressing PtrVCS2 Gene Improves Drought Resistance

**Figure 2:**
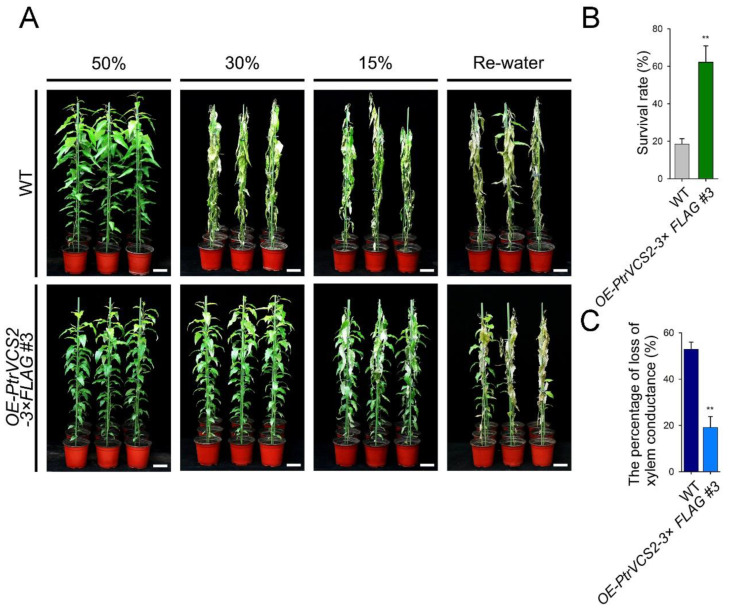
Overexpressing *PtrVCS2* gene improves drought resistance of *P. trichocarpa*. (**A**) Drought resistance phenotypes of wild-type and *OE-PtrVCS2* transgenic plants. Plants (before drought, on the left) were dehydrated by reducing soil water content to 30%, 15% and then rehydrated for 3 days (Rehydrated for 3 days, on the right). Bars = 10 cm. (**B**) Statistical analysis of survival rates after drought treatment and recovery. The average percentage of survival and standard errors were calculated from three independent experiments with at least 12 plants of each genotype in each replicate. (**C**) Percentage loss of xylem conductance (PLC) of wild-type and *OE-PtrVCS2* transgenic plants. In the statistical analysis, the average percentage of PLC was calculated from three independent experiments and standard errors were calculated from three independent experiments. Asterisks indicate significant differences between the *OE-PtrVCS2* transgenics and WT plants. ** *p* < 0.01 (Student’s *t*-test).

Next, we tested the drought response of *OE*-*PtrVCS2-3×FLAG* #3 and wild-type plants. Four-month-old *OE*-*PtrVCS2* and wild-type plants were subjected to drought treatments by withholding water. When plants were adequately watered, the soil was saturated, having 50% volumetric soil water content (VWC) (Figure 2A). In the absence of water replenishment, the VWC decreased to 30%, and the leaves of wild-type plants wilted, while most of the *OE*-*PtrVCS2* transgenics remained turgid (Figure 2A). Under the severe drought condition with 15% VWC, the wild-type plants showed severe wilting symptoms, compared with the *OE*-*PtrVCS2* transgenics (Figure 2A). After rehydration in 3 days, the survival rate for the stressed plants was estimated (Figure 2A). Most of the wild-type plants did not recover, with an 18.4% survival rate (Figure 2B). By contrast, the *OE*-*PtrVCS2* transgenics recovered rapidly, with a survival rate of 62.2% (Figure 2B).

Although the leaves of the *OE*-*PtrVCS2* plants were smaller than those of the wild-type plants, the number of functional leaves of the transgenics were much greater than those of the wild-type plants under drought stress conditions (Supplemental Figure S1). We measured PLC in the stems of the *OE-PtrVCS2* and wild-type plants. Under the drought condition with 15% VWC, the *OE-PtrVCS2* transgenics have lower PLC (19.1%, Figure 2C) compared with wild-type plants (52.9%, Figure 2C).

### 2.3. PtrVCS2 Regulates Stomatal Closure-Related Genes

**Figure 3:**
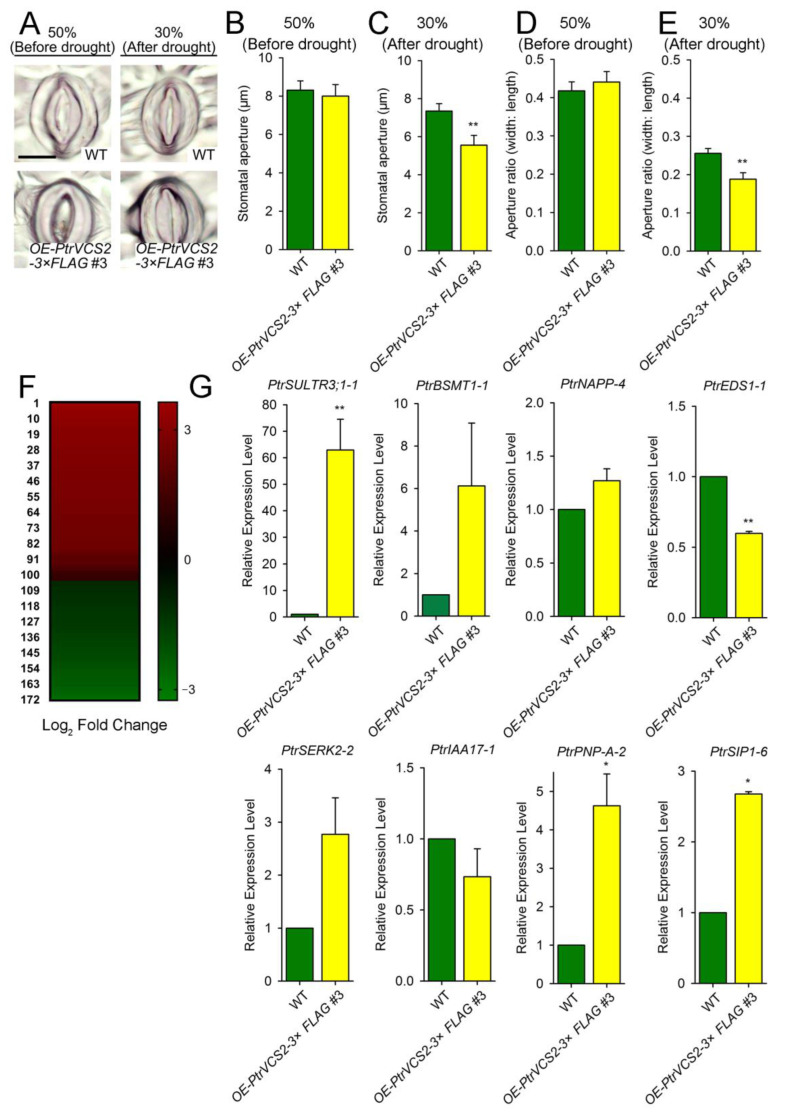
The stomata aperture of the wild-type and *OE-PtrVCS2* plants in response to drought stress. (**A**) Photographs of stomata on the leaves of the wild-type (WT) and *OE-PtrVCS2* plants before or after drought stress treatments. Bars = 20 μm. (**B**) The stomatal aperture under the condition with 50% VWC (before drought stress treatments). (**C**) The stomatal aperture under the condition with 30% VWC (after drought stress treatments). (**D**) The aperture ratio under the condition with 50% VWC (before drought stress treatments). (**E**) The aperture ratio under the condition with 30% VWC (after drought stress treatments). (**F**) Identification of differentially expressed genes in the *OE-PtrVCS2* transgenics under the drought condition. (**G**) The relative expression levels of the genes related to stomata closure in the leaves of the WT and *OE-PtrVCS2* plants under the drought condition. The error bars represent one SE of the three biological replicates. “*”,” **” denote significant differences: * *p* < 0.05, ** *p* < 0.01 (Student’s *t*-test).

Stomatal movement experiments revealed that the *OE-PtrVCS2* transgenics showed lower stomata aperture than wild-type plants under the drought condition with 30% VWC (Figure 3A–E). These results suggested that overexpression of *PtrVCS2* may improve drought resistance by regulating stomatal aperture.

To explore the molecular regulation of *PtrVCS2* in drought resistance of *P. trichocarpa* plants, we performed RNA-seq analysis of WT and *OE-PtrVCS2* plants with stem-developing xylem (SDX) tissues under the drought condition with 30% VWC (Figure 2). We identified 172 differentially expressed genes (DEGs) in the *OE-PtrVCS2* transgenics with 103 up-regulated and 69 down-regulated genes (Figure 3F) (Supplemental Dataset S1). Of these DEGs, the expression levels of multiple genes involved in regulation of stomatal opening and closing were obviously changed (Supplemental Table S1). The expression levels of these stomatal closure-related genes were analyzed in leaves of WT and *OE-PtrVCS2* plants with drought treatments. We found that the expression levels of five genes, whose homologous genes such as *PtrSULTR3;1-1*, *PtrNAPP-4*, *PtrEDS1-1*, and *PtrSERK2*-2 which have functions in promoting stomatal closure, were highly induced by drought stress (Figure 3G). Considering that induction by drought stress is particularly apparent in *PtrSULTR3;1-1*, we speculated that *PtrVCS2* may control stomatal closure by regulating the expression of the *PtrSULTR3;1-1* gene in *P. trichocarpa*.

### 2.4. PtrVCS2 Regulates the Genes Related to Cell Wall Biosynthesis

**Figure 4:**
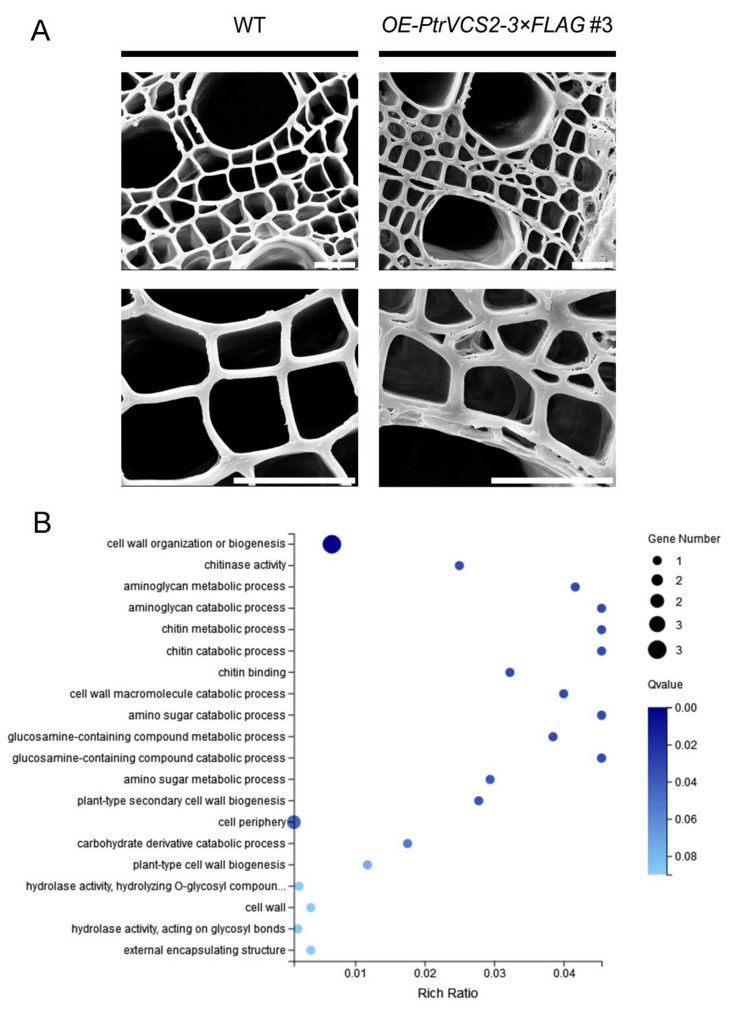
*PtrVCS2* regulates the cell wall biosynthesis-related genes. (**A**) Wild-type (WT) and *OE-PtrVCS2* transgenic plants were imaged by SEM with the 12th internode imaged at ×1000 (upper panels) and ×3000 (lower panels) magnification. Bars = 20 μm. (**B**) Gene ontology categories related to cell wall biosynthesis.

Scanning electron micrographs show that elevated *PtrVCS2* expression resulted in thicker fiber and vessel cell walls in the xylem of the *OE-PtrVCS2* transgenics compared with the WT plants (Figure 4A). Next, we conducted gene ontology (GO) analysis (Figure 4B), with 172 DEGs identified from the RNA-seq of WT and *OE-PtrVCS2* plants under drought conditions, and found three genes: *PtrPR3-3*, *PtrFLA11-12*, and a novel one, related to cell wall organization or biogenesis (Supplementary Table S2). Moreover, we found that *PtrVCS2* regulates three genes, *PtrGH9B1-1*, *PtrLAC44*, and *PtrLAC45* under drought conditions, and their homologous genes function in regulating cellulose and lignin biosynthesis.

### 2.5. Overexpressing PtrVCS2 Gene Improves the Adaptability of P. trichocarpa to Chronic Drought Stress

**Figure 5:**
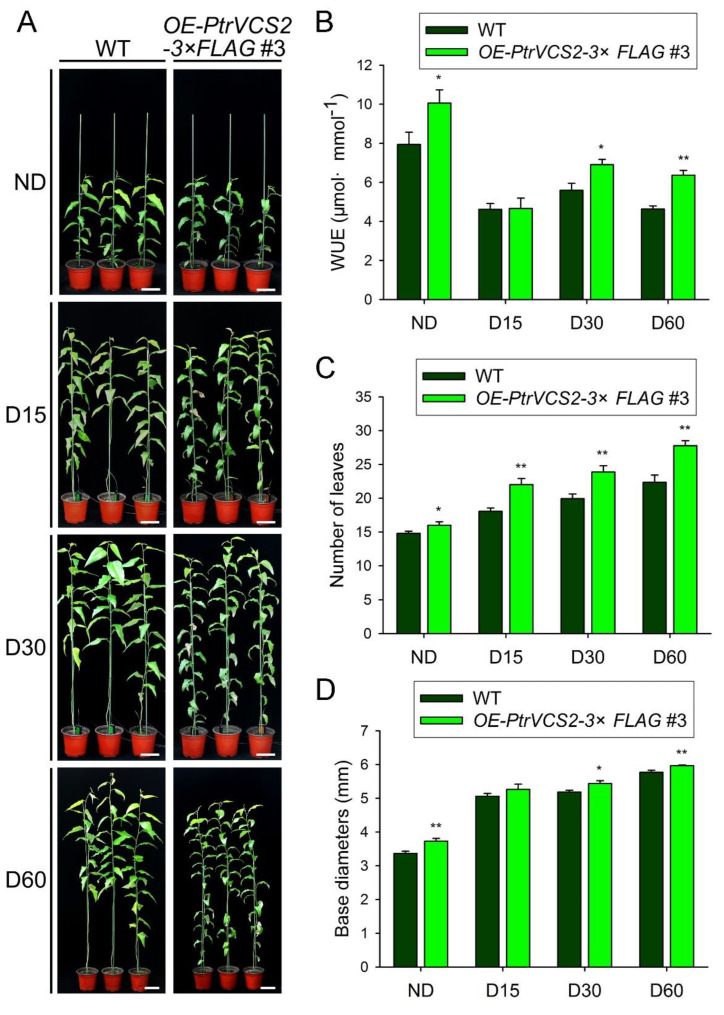
Overexpressing *PtrVCS2* gene improves the adaptability of *P. trichocarpa* to chronic drought stress. (**A**) Phenotypes of the *OE-PtrVCS2* transgenic and wild-type plants with chronic stress treatments. Plants (before drought, on the left) were dehydrated by reducing soil water content to 40% and maintaining it for 60 days. ND (normal condition, 50% VWC), D15 (15-day, 40% VWC), D30 (30-day, 40% VWC), and D60 (60-day, 40% VWC). Bars = 10 cm. (**B**) Water use efficiency of the *OE-PtrVCS2* transgenic and WT plants. (**C**) Number of leaves of the *OE-PtrVCS2* transgenic and WT plants. (**D**) Base diameters of the *OE-PtrVCS2* transgenic and WT plants. The error bars indicate one standard error of three biological replicates from independent pools of *P. trichocarpa* plants. “*”, “**” indicate significant differences between the *OE-PtrVCS2* transgenics and WT plants. * *p* < 0.05, ** *p* < 0.01 (Student’s *t*-test).

Chronic drought, a continuous stress, inhibits the biomass accumulation of plants. To further elucidate the role of *PtrVCS2* in response to drought stress, we employed a chronic water deficit assay (Figure 5A).

Under normal conditions (ND (normal condition, 50% VWC)), the transgenics had higher WUE (10.1 μmol·mmol^−1^) than the WT. After 15-day drought treatments, there was no significant difference in WUE between the *OE-PtrVCS2* transgenics and the WT plants. After 30-day drought treatments, the WUE of the *OE-PtrVCS2* transgenics (6.9 μmol·mmol^−1^) was higher than that of the WT plants (5.6 μmol·mmol^−1^). After 60-day drought treatments, the WUE of the *OE-PtrVCS2* transgenics (6.4 μmol·mmol^−1^) was significantly higher than that of the WT plants (4.6 μmol·mmol^−1^) (Figure 5B). Moreover, the *OE-PtrVCS2* transgenic plants had more leaves (Figure 5C) and larger base diameters (Figure 5D) than the WT plants. These results indicated that overexpressing *PtrVCS2* could improve the adaptability of *P. trichocarpa* to chronic drought stress.

## 3. Discussion

Changes in the duration and frequency of drought led to a gradual increase in tree mortality [36]. Land drought has become a key global problem [37]. Transpiration forces transport water and solutes absorbed from the roots through the vessel cells to the leaves, and the water deficit can cause the water column in vessels to break, thus forming an embolism that blocks water transport in the stem [7,38]. In this study, we identified a gene in *P. trichocarpa*, *PtrVCS2*, which plays a vital role in plant response to drought. Overexpressing *PtrVCS2* in *P. trichocarpa* resulted in reduced growth and enhanced development of more and smaller vessel cells (Figure 1), which promoted the ability of plants to resist embolism (Figure 2C) [19]. The *OE-PtrVCS2* transgenic plants had higher survival rate (Figure 2B) and lower water conductivity loss (Figure 2C) compared with wild-type plants under drought conditions, indicating that overexpression of *PtrVCS2* is conducive to drought adaptation [39,40]. The overexpression plants had a large number of functional leaves after drought stress, suggesting that these transgenics do not require less water than the wild-type plants under drought stress conditions. Moreover, drought-induced stomatal closure of leaves reduced gas exchange and water loss [12], which protected the *OE-PtrVCS2* transgenics from stress.

There is a trade-off between growth and stress responses such that loss of growth and development tends to enhance the stress resistance of plants [41]. Overexpression of *PtrVCS2* gene led to reduced growth phenotype of the transgenic plants (Figure 1A). RNA-seq analysis of the transgenics revealed that two auxin related genes, *PtrIAA17-1* (*Potri.001G177400*) and *PtrIAA4-4* (*Potri.008G161100*), were inhibited by *PtrVCS2* (Supplemental Dataset S1). There is one possibility that the inhibition of *PtrVCS2* on auxin response genes may be the reason for reduced growth of the transgenic plants. *PtrVCS2* encodes a ZF-HD transcription factor, and the homologs of *PtrVCS2* in other species were also found to be involved in regulation of plant development. In soybean, *GhMIF* regulates petal elongation by activating the expression of *GEG* gene of GASA family members [42]. *MIF1* is related to root growth reduction and dwarfism in *Amaranthus hypochondriacus* L. [43]. Recently, we demonstrated that *PtrVCS2* regulates cambium development by directly binding to *PtrWOX4a* gene promoter through interaction with *PtrWOX13* in *P. trichocarpa* [34]. In this study, we found that *PtrVCS2* not only reduces the number of cambium layers but also inhibits the enlargement of xylem vessel cells. The individual vessel cell area was smaller in the *OE-PtrVCS2* transgenics than in wild-type plants (Figure 1C). Small vessels are less prone to embolism [17]; therefore, the loss of xylem conductance of *OE-PtrVCS2* transgenics was lower than that of the WT (Figure 2C). Reduction in the size of the vessel is one approach to improve WUE. The vessel diameter correlated negatively with intrinsic WUE, and the correlation was stronger under water deficit conditions [44]. We speculate that WUE of *OE-PtrVCS2* transgenics was also higher than that of WT under drought stress. Although smaller vessels have little embolism, they also transport less water, which is not sufficient for the growth of the plants [4]. We found that the vessel cell number (Figure 1D) and the area of vessels in the transverse section of the woody stem (Figure 1E) were significantly increased in the *OE-PtrVCS2* transgenics compared with wild-type plants. The increase in the number of vessels solves the problem of water loss caused by the smaller area of the individual cells in *OE-PtrVCS2* transgenic plants. Moreover, we demonstrated that *PtrVCS2* plays a positive role in improving drought adaptability and resistance in *P. trichocarpa* (Figure 2 and Figure 5). Previous studies and our findings suggest that *PtrVCS2* and its homologs in different species have diverse functions not only in the regulation of plant development but also in plant response to abiotic stresses.

Plant growth adapts to the changing environments. Under drought conditions, plants alter their physiology to reduce growth and enhance drought resistance for adaptation [39,45,46,47,48]. Drought adaptation also includes development of cell wall structures that are conducive to water transport, i.e., structures with smaller stem xylem vessels to minimize xylem cavitation (failure of upward water transport) [49,50]. Cell walls limit cells to different shapes and protect the cells in response to abiotic stresses [51,52]. The secondary cell wall acts as a conduit for water and resistance to the tension generated by the transpiration pull [53]. We observed the cross section of the stem by scanning electron microscope and found that the xylem cell wall of the *OE-PtrVCS2* plants was thicker than that of the wild-type plants (Figure 4A). Therefore, it is possible that *PtrVCS2* may restrict xylem cell morphological changes by regulating cell wall thickening. Moreover, *PtrVCS2* regulated the expression of the cell wall synthesis genes, such as *PtrFLA11-12* and *PtrPR3-3* (Figure 4B). The homologous gene of *PtrFLA11-12* is associated with cell wall expansion [54], and the homologous gene of *PtrPR3-3*, as a marker gene of *Botrytis cinerea*, has been shown to promote disease resistance in plants [55]. In *Arabidopsis thaliana*, *AtGH9B1-1* is involved in cellulose biosynthesis [56]. In *Cleome hassleriana* L., *LACs* promote the biosynthesis of lignin components [57]. Together, these results suggest that *PtrVCS2* may control xylem cell morphological changes by regulating the expression of these cell wall biosynthesis-related genes in *P. trichocarpa*. The increase of cell wall thickness increases the density of wood, thus changing the wood properties and increasing the biomass of plants [53,58]. The key traits in the *OE-PtrVCS2* transgenics, i.e., reduced plant growth and a larger number of small xylem vessels that are consistent with overexpression of *PtrVCS2*, are all conducive to drought adaptation. The recovery of plant stems with small and abundant cells and thick cell walls may depend on the recovery of xylem function and the regulation of carbohydrate metabolism in xylem after rehydration after drought stress [59]. These apparent adaptative traits make the *OE-PtrVCS2* lines unique as potential sources for the attainment of new knowledge of the complex regulations involved in cell-type biosynthesis in wood formation and growth under stresses.

Hydraulic conductivity is positively correlated with stomatal conductance, and a decrease in water transport leads to stomatal closure [60,61]. In response to drought stress, plants use hormones to trigger stomatal closure, reducing water loss [62]. Using transgenesis (Figure 2) and RNA-seq (Figure 3) we found that *PtrVCS2* controls stomatal closure by regulating the expression of stomatal closure-related genes in plant response to drought (Supplemental Table S1) under drought conditions [63,64,65,66,67,68,69,70]. Of these genes, the expression levels of *PtrSULTR3; 1-1* were significantly increased in xylem and leaves of the *OE-PtrVCS2* transgenic plants compared with wild-type plants under drought conditions (Figure 3G). The homolog of *PtrSULTR3;1-1* in *Arabidopsis*, *AtSULTR3,* moves into chloroplasts via sulfate transport to promote cysteine synthesis, trigger ABA biosynthesis, and regulate stomatal closure under stress conditions [63]. The most stomata of the *OE-PtrVCS2* transgenic plants were closed under drought stress (Figure 3A,E), suggesting that *PtrVCS2* may regulate the expression of these stomatal closure-related genes and thus control water losses through transpiration of the leaves by an ABA-dependent pathway. Additionally, the expression levels of *PtrPNP-A-2* were also induced by *PtrVCS2*, but the relative expression abundance was lower than that of *PtrSULTR3; 1-1* (Figure 3G). The homologue of *PtrPNP-A-2* in *Arabidopsis*, *AtPNP-A*, as an antagonist of ABA, limits gas exchange in stomata [66]. There is one possibility that *PtrSULTR3; 1-1* and *PtrPNP-A-2* may play opposite roles in ABA-induced stomatal closure, but we do not know when *PtrPNP-A-2* is dominant. Usually, ABA signaling receptors inhibit PP2C phosphatase activity and promote the activation of SnRK2s protease phosphorylation to trigger stomatal closure [71]. Whether *PtrVCS2* is involved in this process, what role it plays, and the target of phosphorylation still needs to be further investigated.

Moreover, gene ontology (GO) analysis showed that *PtrVCS2* regulates the expression of genes related to the ABA signaling pathway in xylem (Supplemental Figure S2). The ZF-HD transcription factor also responded to abiotic stress and the ABA hormone signal in tomato [72]. Thus, *PtrVCS2* may control stomatal closure to improve drought resistance by integrating ABA signals in *P. trichocarpa*. Further exploration of the connections between the *PtrVCS2*-mediated control of stomatal closure and ABA signaling should yield new insights into the regulation of drought resistance in *P. trichocarpa*. In addition to the ABA signaling pathway, *PtrVCS2* also regulates the expression of the BR response gene *PtrCYP67A2*-13 (*Potri.009G064800*) and the ethylene response gene *PtrPR4-3* (*Potri.013g041600*) (Supplemental Dataset S1). Considering the complexity of the regulatory network of plant stress response [73,74], it is possible that multiple hormone signaling pathways are involved in improving drought resistance of *OE-PtrVCS2* plants.

Water deficit often results in the decrease of aboveground biomass and retardation of plant growth [75]. Under chronic drought stress, the leaves of wild plants fall off (Figure 5A), but there was no obvious leaf loss in *OE-PtrVCS2* plants. The number of leaves of *OE-PtrVCS2* transgenics was significantly more than that of wild type plants (Figure 5C). WUE is a key ecosystem attribute of plant water cycles [76]. The *OE-PtrVCS2* transgenic plants had strong photosynthesis and high water use efficiency under drought conditions (Supplemental Figure S3), indicating that *OE-PtrVCS2* plants produced more biomass for the same amount of water consumption [77]. This is consistent with the increase in stem size and wood yield of *OE-PtrVCS2* transgenics (Figure 5D). We demonstrated that the *OE-PtrVCS2* transgenics could maintain high WUE (Figure 5B) and thus accumulate a large amount of biomass under chronic drought stress condition.

In conclusion, we present a novel gene, *PtrVCS2*, in regulation of secondary xylem development and drought resistance. Knowledge of *PtrVCS2* regulation may help design genetic controls that could maximize beneficial wood traits while minimizing negative effects on growth under drought conditions.

## 4. Materials and Methods

Nisqually-1, a genotype of *P. trichocarpa* Torr. & A. Gray ex. Hook., was used for all experiments with *P. trichocarpa* genotype. Wild-type and transgenic plants were grown in a greenhouse as previously described [39]. *PtrVCS2 (Potri.004G126600)* was identified as a high-expression gene in the stem from RNA-seq data [39]. Amino acid sequences of *PtrVCS2* and other homologous genes mentioned in the article were obtained from the Phytozome, a comparative platform for green plant genomics (https://phytozome-next.jgi.doe.gov/, accessed on 25 August 2021), and conserved motifs were identified in NCBI (https://www.ncbi.nlm.nih.gov/Structure/cdd/wrpsb.cgi, accessed on 8 October 2021). The full CDS of the *PtrVCS2* gene was cloned into pENTR/D-TOPO vector (Invitrogen), and then a pBI121-35Spro-*PtrVCS2-3×FLAG* plant expression vector was generated. Transgenic plants were obtained by using the *P. trichocarpa* transformation system [78]. Three independent transgenic lines, *OE*-*PtrVCS2-3×FLAG* #1, *OE*-*PtrVCS2-3×FLAG* #2, *OE*-*PtrVCS2-3×FLAG* #3 were generated as previously described [34].

### 4.1. RNA-Seq Assay and Data Analyses

In RNA-seq experiments, 4-month-old wild-type *P. trichocarpa* plants and *OE*-*PtrVCS2* transgenic plants were placed in a separate 15-cm pot, which were used for drought treatments (30% VWC). The plants were divided into two groups: (1) control (WT), and (2) *OE*-*PtrVCS2* transgenic plants (OE). Before drought treatment, all plants were thoroughly watered until the soil was saturated. Short-term drought treatments were conducted according to established procedures [39] with a few modifications. Plants in saturated soil were placed in a greenhouse to stand, without replenishing water, and soil water content was reduced to 30%. All the plants were harvested at 30% VWC. Stem-developing xylem tissues for RNA-seq were packed in aluminum foil and collected in liquid nitrogen [79,80]. Total RNA was extracted using the Qiagen RNeasy Mini Kit (Qiagen, Hilden, NRW, Germany) as previously described [81]. RNase-Free DNase Set (Qiagen) was used to remove DNA impurities. Purified RNA was used for RNA-seq. RNA-seq library construction of each sample was performed using the Illumina TruSeq RNA sample preparation kit. The quality of libraries was examined using a Bioanalyzer 2100 (Agilent, Santa Clara, CA, USA). Six libraries were sequenced using the Illumina Genome Analyzer, with an average reading length of 100-bp. After the library index sequences from each read were removed by HISAT [82] with parameters (--dta --phred64 unstranded --new-summary -x index -1 read_r1 -2 read_r2 (PE)), the remaining RNA-seq reads were mapped to the *P. trichocarpa* genome v.4.0. The frequency of raw counts was determined by DIAMOND tools [83,84,85] for all annotated genes. DEGs between the WT and OE samples were identified using DEseq2 [86,87] based on raw counts of mapped RNA-seq reads to annotated genes. Gene ontology (GO) was analyzed using R (3.1.2) statistical software with the Phyper function by Fisher’s exact test with FDR multiple test correction (FDR ≤ 0.05).

### 4.2. RT-qPCR Analyses

Mature leaves of wild-type and *OE-PtrVCS2* transgenics after drought stress treatments were harvested for RNA extraction. RNase-Free DNase Set (Qiagen) was used to remove DNA. Purified RNA was stored in the refrigerator at −80 °C. cDNAs were synthesized by reverse transcription using SrimeScript™ RT reagent Kit (TAKARA) according to the manufacturer’s protocol. FastStart Universal SYBR Green Master (Roche) was used to formulate the reaction system and an Agilent Mx3000P Real-time PCR System was used for the RT-qPCR experiment. Primers for RT-qPCR are listed in Supplemental Table S3.

### 4.3. Histochemical and Histological Analyses

The 5th–10th internodes of *OE-PtrVCS2* transgenics and wild-type plants were harvested and cut into 2–3 mm fragments, and then immersed in 4% formaldehyde following established procedures [39]. The cut wax tape was fixed on the slide and immersed in 2.5% safranin O and 1.25% fast green. The size and area of vessels were measured by using PreciPoint Streaming Software of Precipoint Digital Scanning microscope imaging System (M8). The observation and statistical methods of stomata were conducted as previously described [88]. The images were scanned with ViewPoint Light. IBM SPSS Statistics 19 was used for statistical analyses.

### 4.4. Scanning Electron Micrograph (SEM) Analyses

Fresh stem segments from the 12th internode of *OE-PtrVCS2* transgenic and WT plants were plated with gold (Au) for 60 s at 10 mA. Fresh leaves of *OE-PtrVCS2* transgenic and WT plants were harvested, and the lower epidermis was coated by nail polish. The leaves were then placed on ice to keep fresh. The leaves were cut into small squares, about 1 cm^2^ in area, for observation of the stomatal aperture. The samples were observed under high vacuum at 15 kV using a Nanotech JCM-5000 [89].

### 4.5. Physiological Index Measurement

Four time points (ND (normal condition, 50% VWC), D15 (15-day, 40% VWC), D30 (30-day, 40% VWC), and D60 (60-day, 40% VWC)) were selected to test key photosynthesis indicators and calculate WUE of the WT and *OE-PtrVCS2* transgenics. At least 12 transgenics and 12 wild-type plants were used for each physiological index measurement. Three independent experiments were carried out. Net photosynthetic rate (Pn) and transpiration rate (Tr) were measured by Yaxin-1102G Portable photosynthesis system. Water use efficiency (WUE) = Pn/Tr × 100%. The base internode was used for measuring the percentage of loss of xylem conductance by XYL’EM- *Plus* (Bronkhorst). Volumetric soil water content (VWC, %) was measured by TDR 350 Soil Moisture Meter (Spectrum).

### 4.6. Drought Treatments

For short-term drought treatments, 4-month-old plants in saturated soil (50% VWC) were placed in a greenhouse without replenishing water, and soil water content was reduced from 50% to 30% to 15% and then finally rehydrated for 3 days. At least 12 transgenic plants and 12 wild-type plants were used for each drought treatment. Three independent experiments were performed. The screening experiments revealed that 15% VWC caused shoot dieback to 4-month-old wild-type *P*. *trichocarpa* plants; this was used for the estimation of the survival rate. After the soil water content was reduced to 15% VWC, all plants were rehydrated for 3 days to estimate their survival rates. For chronic drought treatments, 3-month-old *OE-PtrVCS2* and WT plants were subjected to drought treatments by withholding water. After 12-day drought treatments, the VWC was reduced to 40% and maintained for 60 days [90].

## Data Availability

The data supporting the findings of this study are available within the article and its Supplemental Information files.

## Supplementary Materials

The following are available online at https://www.mdpi.com/article/10.3390/ijms24054458/s1.
