# Supplementary material for: PtrVCS2 Regulates Drought Resistance by Changing Vessel Morphology and Stomatal Closure in Populus trichocarpa
Source: Int J Mol Sci. 2023 Feb 24;24(5):4458. doi: 10.3390/ijms24054458 (PMC10003473; doi:10.3390/ijms24054458)
Supplement: Supplementary file 1 [file ijms-24-04458-s001.zip › ijms-2185697-Supplementary Tables and Figures.pdf]

Supplemental Table S1. The differentially expressed genes related to stomata closure under drought stress.

Gene ID	Common Name	WT FPKM	*OE-PtrVCS2* FPKM	Fold Change	References
Potri.002G049500	*PtrSULTR3;1-1*	1.9	71.77	3.44	(Chen et al., 2019)
Potri.007G021300	*PtrBSMT1-1*	0.32	18.45	3.35	(Zheng et al., 2012)
Potri.015G123900	*PtrNAPP-4*	0.03	1.06	2.76	(Huang et al., 2019)
Potri.006G179300	*PtrPNP-A-2*	16.06	213.79	2.62	(Wang et al., 2011)
Potri.015G069600	*PtrEDS1-1*	0.14	0.78	2.06	(Zheng et al., 2015)
Potri.007G082300	*PtrSERK2-2*	1.1	6.77	1.95	(Meng et al., 2015)
Potri.001G177400	*PtrIAA17-1*	176.87	87.84	-1.05	(Balcerowicz et al., 2014)
Potri.017G036700	*PtrSIP1-6*	24.78	7.08	-1.76	(Xu et al., 2018)

Supplemental Table S2. The differentially expressed genes related to cell wall biosynthesis under drought stress.

**Gene ID**	**Common Name**	**WT FPKM**	***OE-PtrVCS2* FPKM**	**Fold Change**	**GO Terms**
Potri.019G088600	*PtrLAC44*	0.25	7.98	3.05	Lignin
Potri.019G088800	*PtrLAC45*	0.64	17.67	2.73	Lignin
Potri.009G141700	*PtrPR3-3 (B-CHI)*	0.64	7.74	2.49	Cell wall
Potri.001G141801	Novel gene	8.93	2.35	-1.77	Cell wall
Potri.019G121100	*PtrFLA11-12*	360.1	88.34	-1.82	Cell wall
Potri.008G132700	*PtrGH9B1-1 (CEL1)*	8.56	1.82	-2.18	Cellulose

Supplemental Table S3. Primer list.

Gene ID (Common name)	Primer Name	Primer sequence (5′- 3′)
Potri.019G010400	RT-*PtrActin*-F	TGTTGCCCTTGACTATGAGCAGGA
(*PtrActin*)	RT-*PtrActin*-R	ACGGAATCTCTCAGCTCCAATGGT
Potri.002G049500	RT-*PtrSULTR3;1-1*-F	GTCCACTAATGTAGAGCGTACACC
(*PtrSULTR3;1-1*)	RT-*PtrSULTR3;1-1*-R	TGAAGTGGTCTGGTTCTTGAATTGT
Potri.007G021300	RT-*PtrBSMT1-1*-F	GAGACAAGTTATGCACAGAACTCAC
(*PtrBSMT1-1*)	RT-*PtrBSMT1-1*-R	GAACATCCCAAGTCTGCTATGGC
Potri.015G123900	RT-*PtrNAPP-4*-F	TCTTACTCATGGTATTCAAGAAGTT
(*PtrNAPP-4*)	RT-*PtrNAPP-4*-R	GTGGCCGACCCAGTAAGCTGC
Potri.006G179300	RT-*PtrPNP-A-2*-F	ATCTCCGTTGCGCATGCTGCAC
(*PtrPNP-A-2*)	RT-*PtrPNP-A-2*-R	ATAACTCCATCGTTCCTGTTTCCG
Potri.015G069600	RT-*PtrEDS1-1*-F	CTGAAGGGATTCATAGTTCATCATC
(*PtrEDS1-1*)	RT-*PtrEDS1-1*-R	GAAAGAACTGAAGATCCACCTTCA
Potri.007G082300	RT-*PtrSERK2*-2-F	GAGAGAGTTTAGGACTTGTCTTGC
(*PtrSERK2*-2)	RT-*PtrSERK2*-2-R	CCATGATATTGTTAGGATCTTGCAG
Potri.001G177400	RT-*PtrIAA17-1*-F	GGTTCAAACTTGCTGGAATCCGAT
(*PtrIAA17-1*)	RT-*PtrIAA17-1*-R	CTCTATTAATGCTAGCCCACGAG
Potri.017G036700	RT-*PtrSIP1-6*-F	TGATAATATAACCCTCAGTCCCTCG
(*PtrSIP1-6*)	RT-*PtrSIP1-6*-R	CTTCAATTTCCCAATATGGACAACG

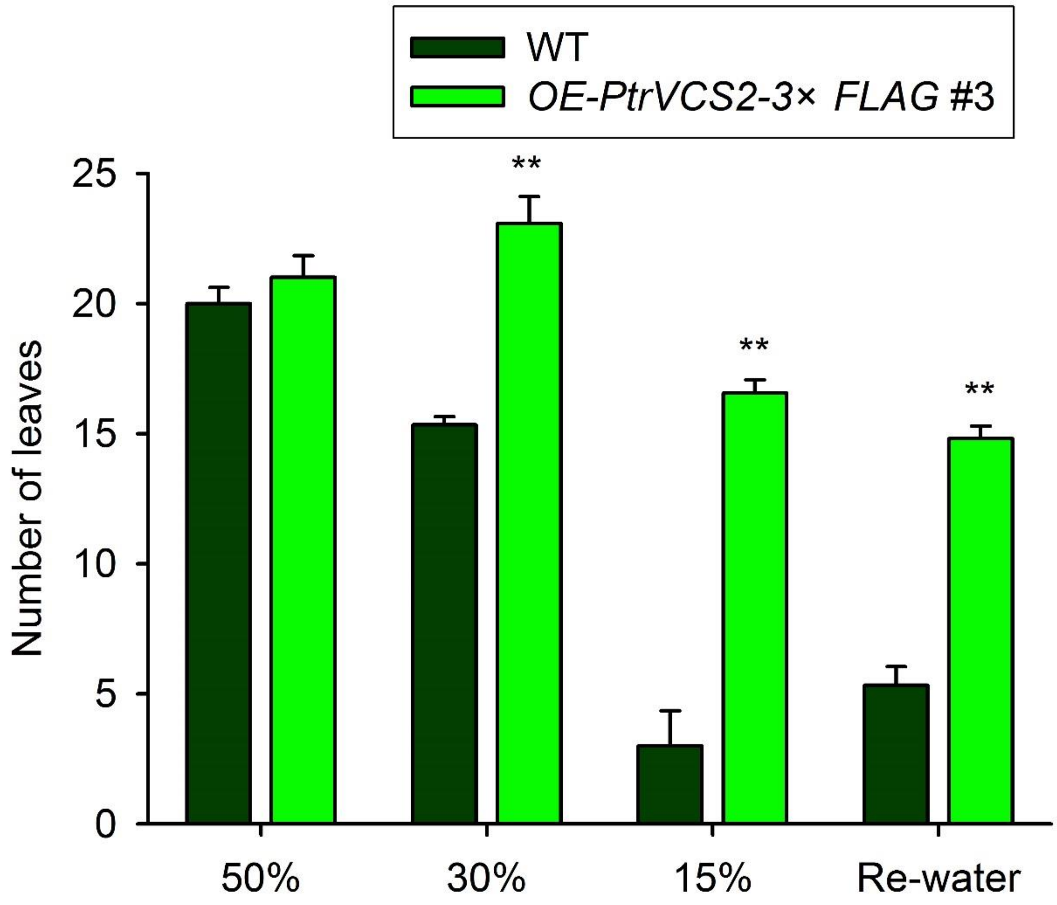

Supplemental Figure S1. Number of functional leaves of the *OE-PtrVCS2* transgenic and wild-type plants under drought stress conditions. ** $p < 0.01$ (Student's *t*-test).

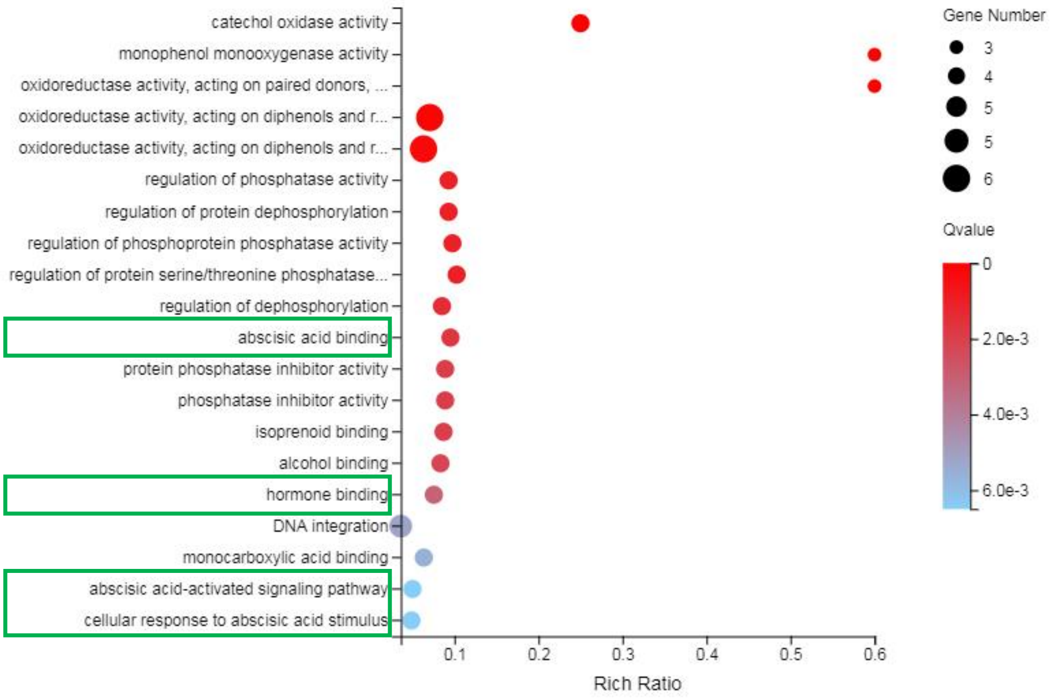

Supplemental Figure S2. The differentially expressed genes related to ABA response under drought stress. Green box: the differentially expressed genes related to ABA signal and other hormones.

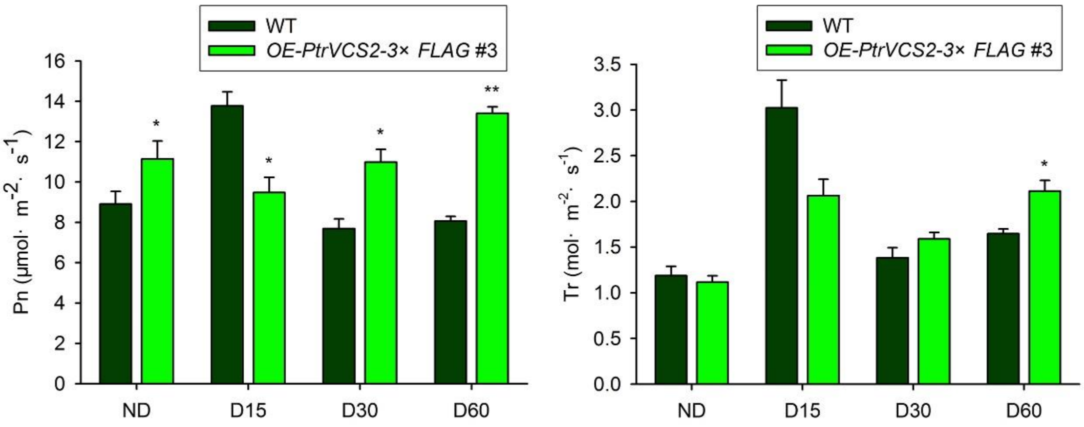

Supplemental Figure S3. Key photosynthesis indicators of *Populus trichocarpa* under chronic drought stress. Net photosynthetic rate (Pn) and transpiration rate (Tr) of the wild-type (WT) and *OE-PtrVCS2* plants. Error bars indicate one SE of three biological replicates from independent pools of *P. trichocarpa* leaves. Asterisks indicate significant differences between the transgenics and WT plants under drought condition. *p <0.05, **p <0.01 (Student's t-test).

Reference

Balcerowicz, M., Ranjan, A., Rupprecht, L., Fiene, G., & Hoecker, U. (2014). Auxin represses stomatal development in dark-grown seedlings via Aux/IAA proteins. *Development (Cambridge, England)*, *141*(16), 3165-3176. https://doi.org/10.1242/dev.109181

Chen, Z., Zhao, P.-X., Miao, Z.-Q., Qi, G.-F., Wang, Z., Yuan, Y., Ahmad, N., Cao, M.-J., Hell, R., Wirtz, M., & Xiang, C.-B. (2019). *SULTR3s* Function in Chloroplast Sulfate Uptake and Affect ABA Biosynthesis and the Stress Response. *Plant Physiology*, *180*(1), 593-604. https://doi.org/10.1104/pp.18.01439

Huang, L., Chen, L., Wang, L., Yang, Y., Rao, Y., Ren, D., Dai, L., Gao, Y., Zou, W., Lu, X., Zhang, G., Zhu, L., Hu, J., Chen, G., Shen, L., Dong, G., Gao, Z., Guo, L., Qian, Q., & Zeng, D. (2019). A Nck-associated protein 1-like protein affects drought sensitivity by its involvement in leaf epidermal development and stomatal closure in rice. *The Plant Journal : For Cell and Molecular Biology*, *98*(5), 884-897. https://doi.org/10.1111/tpj.14288

Meng, X., Chen, X., Mang, H., Liu, C., Yu, X., Gao, X., Torii, K. U., He, P., & Shan, L. (2015). Differential Function of Arabidopsis SERK Family Receptor-like Kinases in Stomatal Patterning. *Current Biology : CB*, *25*(18), 2361-2372. https://doi.org/10.1016/j.cub.2015.07.068

Wang, Y. H., Gehring, C., & Irving, H. R. (2011). Plant natriuretic peptides are apoplastic and paracrine stress response molecules. *Plant & Cell Physiology*, *52*(5), 837-850. https://doi.org/10.1093/pcp/pcr036

Xu, H., Shi, X., He, L., Guo, Y., Zang, D., Li, H., Zhang, W., & Wang, Y. (2018). *Arabidopsis thaliana* Trihelix Transcription Factor AST1 Mediates Salt and Osmotic Stress Tolerance by Binding to a Novel AGAG-Box and Some GT Motifs. *Plant & Cell Physiology*, *59*(5), 946-965. https://doi.org/10.1093/pcp/pcy032

Zheng, X.-Y., Spivey, N. W., Zeng, W., Liu, P.-P., Fu, Z. Q., Klessig, D. F., He, S. Y., & Dong, X. (2012). Coronatine promotes Pseudomonas syringae virulence in plants by activating a signaling cascade that inhibits salicylic acid accumulation. *Cell Host & Microbe*, *11*(6), 587-596. https://doi.org/10.1016/j.chom.2012.04.014

Zheng, X.-Y., Zhou, M., Yoo, H., Pruneda-Paz, J. L., Spivey, N. W., Kay, S. A., & Dong, X. (2015). Spatial and temporal regulation of biosynthesis of the plant immune signal salicylic acid. *Proceedings of the National Academy of Sciences of the United States of America*, *112*(30), 9166-9173. https://doi.org/10.1073/pnas.1511182112
